# The rs7911488-T allele promotes the growth and metastasis of colorectal cancer through modulating miR-1307/PRRX1

**DOI:** 10.1038/s41419-020-02834-x

**Published:** 2020-08-07

**Authors:** Man Yang, Xinchang Liu, Fanyi Meng, Yawen Zhang, Mengmeng Wang, Yinshuang Chen, Xuqin Guo, Weichang Chen, Weipeng Wang

**Affiliations:** 1 Center for Drug Metabolism and Pharmacokinetics, College of Pharmaceutical Sciences, Soochow University, Suzhou, 215123 China; 2 Jiangsu Key Laboratory of Clinical Immunology, Soochow University, Suzhou, 215006 China; 3 Jiangsu Key Laboratory of Gastrointestinal Tumor Immunology, The First Affiliated Hospital of Soochow University, Suzhou, 215006 China

**Keywords:** Cancer genetics, Oncogenesis

## Abstract

We previously discovered that rs7911488T>C in pre-miR-1307 was closely correlated to the risk of colorectal cancer (CRC). However, the roles of rs7911488 in CRC are still largely unknown. Here we explored the roles of rs7911488 in the growth and metastasis of CRC. We firstly generated cell lines SW480-T and SW480-C for stable expression of rs7911488 T-allelic and C-allelic pre-miR-1307, respectively. We subcutaneously grafted the cells into nude mice. We found that SW480-T tumors with high expression of miR-1307 obviously grew faster than the SW480-C tumors. Moreover, liver metastases (5/8) were observed in the mice bearing SW480-T tumors but not the SW480-C tumor-bearing mice. The results from colony formation assays, transwell assays, and wound healing assays demonstrated that the proliferative and metastatic abilities of SW480-T cells were evidently more potent than the SW480-C cells. Then we utilized gene array, real-time PCR, western blotting, and dual-luciferase reporter assays to figure out that miR-1307 directly inhibited PPRX1 expression by binding to its 3′-UTR. Thereafter, we confirmed that the proliferative and metastatic abilities of SW480 and HCT-116 cells were markedly enhanced by miR-1307, but were suppressed by PRRX1. Moreover, the regulatory roles of miR-1307 in the proliferation and metastasis of CRC cells were reversed by PRRX1. Notably, we also found that PRRX1 repressed CRC tumor growth in nude mice. In summary, our current study revealed that rs7911488-T allele led to over-expression of miR-1307, which inhibited PRRX1 and consequently promoted the proliferation and migration of CRC cells. This might offer a novel insight into the progression of CRC.

## Introduction

With increasing incidence and mortality, cancer has become one of the major public health problems^1^. In 2016, the National Cancer Center released the latest cancer statistics, which showed that the incidence and mortality of colorectal cancer (CRC) in China ranked fifth among all malignant tumors^2^. Due to the changes in diets, increased consumption of high-fat foods, genetic mutations, inflammatory bowel disease, and smoking, the incidence and mortality of CRC has increased year-by-year^2^. Benefited from the advancements in cancer treatment, the 5-year survival rate of early-stage CRC patients reached 80%, but only about 50% of patients with middle- or advanced-stage CRC could survive over 5 years^2^. Therefore, elucidating the molecular mechanisms underlying the occurrence and development of CRC is of great significance to develop novel therapeutic strategies.

MicroRNA (miRNA) is a class of single-stranded and noncoding small RNAs with a length of about 19–25 nucleotides. miRNA completely or incompletely binds to the 3′-untranslated region (3′-UTR) of the target gene, resulting in degradation or inhibition of translation of the target gene. Recently, a number of endogenous miRNAs have been identified and each miRNA can regulate several target genes. Moreover, studies have demonstrated that a variety of miRNAs are abnormally expressed in tumors. For instance, high expression of miR-135, miR-494, miR-19a, miR-224, miR-574, and miR-638^3–8^ and low-expression of miR-187, miR-34a, let-7c, miR-145, miR-30a, and miR-384^9–14^ were observed in CRC tissues, which have been confirmed to play critical roles in the occurrence and development of CRC.

Single nucleotide polymorphism (SNP) is the most common genetic variation in human genomic DNA. Due to the rapid development of high-throughput detection technologies, the roles of SNP in tumorigenesis have been widely studied. In case that an SNP locates in the 3′-UTR of a miRNA gene or a target gene, it might disrupt the expression of miRNA or the binding of miRNA to its target gene, resulting in dysregulation of target genes. Numerous studies have reported that miRNA-related SNPs are closely correlated to the occurrence and development of CRC. For instance, CD86 rs17281995 and INSR rs1051690 were associated with the risk of CRC^15^. Rs17281995, locating at CD86 3′-UTR, contributes to the risk of CRC by affecting the regulatory roles of miRNAs including miR-337, miR-582, miR-200a, miR-184, and miR-212^16^. The SNP rs4596 C>G in the 3′-UTR of GTF2H1 was involved in the susceptibility to CRC. Carriers of rs4596-G allele had lower risk of CRC than those of C allele, due to interference of the binding of miR-518a-5p and miR-527 to GTF2H1^17^. The rs2147578 in lnc-LAMC2 led to an increased risk of CRC in a Chinese population through disrupting its binding to miR-128-3p and consequent dysregulation of oncogene LAMC2^18^. Shen et al. found that the rs1317082 T>C in the exon of CCSlnc362 provided a binding-site to miR-4658, resulting in the downregulation of CCSlnc362 and inhibition of the proliferation of CRC cells, and thus reduced the susceptibility to CRC^19^. We previously found that rs7911488 in stem-loop of pre-miR-1307 was also significantly associated with the risk of CRC^20^. The C allele interfered with the normal cleavage of DICER1 by binding to MBNL1, which led to low-expression of miR-1307 and high expression of its target gene BCL2. Furthermore, we found rs7911488 was significantly correlated to the efficacy of capecitabine-treated CRC patients. The C allele caused low-expression of miR-1307 and high expression of TYMS, which reduced the sensitivity of CRC cells to capecitabine chemotherapy^21^.

Several studies have revealed that miR-1307 is closely related to the occurrence and development of cancers. Shimomura et al. found that the expression of miR-1307 was increased in the serum of breast cancer patients, which significantly promoted the proliferation of breast cancer cells^22^. In addition, miR-1307 also promoted the occurrence and development of breast cancer by inhibiting the expression of its target gene SMYD4^23^. Chen et al. provided evidence to support that miR-1307 played a very important role in the progression of liver cancer by targeting DAB2IP^24^. Moreover, studies demonstrated that miR-1307 boosted the progression of prostate cancer by targeting FOXO3A^25^ and induced chemoresistance of ovarian cancer cells by inhibiting ING5^26^.

In this study, we investigated the effect of rs7911488 T>C on the growth and metastasis of CRC. We subcutaneously grafted rs7911488 T-allelic and C-allelic cells into nude mice. We found that the T-allelic tumors had higher expression of miR-1307, faster growth, and more metastases than the C-allelic tumors. Then we identified PRRX1 as a target gene of miR-1307, and we confirmed that miR-1307 promoted the proliferation and migration of CRC cells through inhibiting PRRX1. Moreover, we also proved that PRRX1 suppressed the growth of tumor in vivo.

## Materials and methods

### Cell lines

The SW480, HCT-116, CHO, and HEK293 cells were purchased from the American Type Culture Collection (Manassas, VA, USA) in January 2019 and were authenticated by the company. They were tested for mycoplasma contamination and passaged in our laboratory for fewer than 6 months after resuscitation. The SW480 and CHO cells were cultured in RPMI-1640 medium (Hyclone), and the HCT-116 and HEK293 cells were cultured in DMEM medium (Hyclone), with 10% fetal bovine serum (FBS; Gbico). All cells were incubated at 37 °C in a humidified incubator with 5% CO_2_. Cells in the logarithmic growth phase were used for experiments.

### Construction of cell lines

To construct rs7911488 T-allelic or C-allelic cells, the expression vectors of rs7911488 T-allelic or C-allelic pre-miR-1307^20^ were transfected into SW480 cells and stable clones (SW480-T and SW480-C) were selected by neomycin. The stable clones were confirmed by flow cytometry assays, qPCR, and DNA sequencing. To construct PRRX1-overexpression cells, the PRRX1/pcDNA3.1 (+) expression plasmids (synthesized by GENEWIZ) were transferred into SW480 or HCT-116 cells. The stable clones (PRRX1^oe^ SW480 and PRRX1^oe^ HCT-116) were selected by G418 and were confirmed by WB method.

### Gene array

Total RNAs were extracted from SW480-T and SW480-C cells, and were quantified by NanoDrop ultra-violet spectrometer (Thermo). The RNA integrity was assessed by Bioanalyzer 2100 (Agilent). The sample labeling, microarray hybridization, and washing were performed based on the manufacturer’s standard protocols. Briefly, total RNAs were transcribed to double-strand cDNA, and then were synthesized into cRNA and labeled with Cyanine-3-CTP. The labeled cRNAs were hybridized onto the microarray. After washing, the arrays were scanned by the Scanner G2505C (Agilent). Feature Extraction software (v10.7.1.1, Agilent) was used to analyze array images to get raw data. Genespring (v13.1, Agilent) was employed to finish the basic analysis with the raw data. The raw data were normalized with the quantile algorithm. The probes that at least 100% of the values in any 1 out of all conditions have flags in “Detected” were chosen for further data analysis.

### Quantitative real-time PCR (qPCR)

SW480 and HCT-116 cells were transfected with miR-1307 mimics or scrambled miRNA (GenePharma) for 48 h. Total RNAs from SW480-T, SW480-C, SW480, and HCT-116 cells were isolated by using TRIzol (Invitrogen) according to the manufacturer’s instructions and subjected to reverse transcription with M-MuLV reverse transcriptase (MBI) and random primer. QPCR was conducted in 20-μl reaction contained 400 nM of each primer (Table S1) and quantitative RT-PCR master mix (Takara). The real-time PCR was conducted on the CFX96 Touch^TM^ real-time PCR system (Bio-Rad). The relative expression was calculated by using the comparative threshold cycle (*C*_t_) method with the formula 2^−∆∆Ct^ and a single threshold for all samples. Each assay was performed in triplicate.

### Western blotting (WB)

Total proteins from the cells transfected with miR-1307 mimics or inhibitors, or PRRX1-expression plasmid or siRNA for 72 h were lysed using RIPA buffer (Beyotime) with the complete protease inhibitor cocktail (Beyotime). The protein concentration was determined using the Pierce BCA Protein Assay Kit (Thermo). Then the proteins were separated on SDS-PAGE gel and electro-transferred onto PVDF membranes (Bio-Rad). The membranes were incubated with PRRX1 (ImmunoWay), GAPDH (Santa Cruz), or β-actin (Beyotime) monoclonal antibody, followed by the secondary HRP-conjugated anti-rabbit antibody or anti-mouse antibody (Beyotime). After the band was washed with 10× Tris-buffered saline Tween (TBST), the proteins on the membranes were visualized with Clarity Western ECL substrates (Bio-Rad) in ChemiDocTM MP Imaging System (Bio-Rad). Each assay was performed in duplicate.

### Dual-luciferase reporter assay

The pGL-3 constructs containing PRRX1 3′-UTR were generated by amplifying the 3′-UTR with the primers (5′-GAC TAG TCT AGA AAT GTT CCT CCT CCC TCT G-3′ and 5′-CTA GCC GTT AAC GAA TAG AGC CTC ACA ACA CC-3′ for the upstream; 5′-GAC TAG TCT AGA CTC TGT ATG TCC AGC ACT TTG-3′ and 5′-CTA GCC GTT AAC CCT GCC AAG TAC CCT ACA AAT-3′ for the downstream). The amplification products were cloned into the downstream of the pGL3-Control vectors (Promega) using *Xba*I and *Hpa*l endonucleases (Thermo). Positive clones were selected by sequence-specific PCR, restriction enzymes digestion, and DNA sequencing method. Then the 3′-UTR/pGL3 constructs were co-transfected with miR-1307 mimics into CHO, HEK293, or SW480 cells by using lipofectamine 2000 (Invitrogen). After incubating for 24 h, the activities of luciferase in cells were detected by using the dual-luciferase reporter assay system (Promega). Each assay was performed in triplicate.

### Colony formation

SW480 and HCT-116 cells were respectively transfected with miR-1307 mimics, miR-1307 inhibitors, PRRX1-expression plasmid, or PRRX1 siRNA for 48 h. Then the SW480-T, SW480-C, SW480, and HCT-116 cells were respectively seeded in 6-well plates at a density of 500 cells per well and incubated for 2 weeks. Next, the cells were fixed by 0.4% paraformaldehyde (Sinopharm Chemical Reagent) and were stained by 0.1% crystal violet (Solarbio Science & Technology) for 30 min. Colonies containing more than 50 cells were counted by using a microscope (Olympus). Each assay was performed in triplicate.

### Wound healing assay

A wound healing assay was performed to examine the migration ability of cells. Briefly, SW480 and HCT-116 cells were respectively transfected with miR-1307 mimics, miR-1307 inhibitors, PRRX1-expression plasmid, or PRRX1 siRNA for 48 h. Then the SW480-T, SW480-C, SW480, and HCT-116 cells were respectively seeded in 24-well plates. When the cells were at 90–95% confluence, a single scratch wound was generated using a 200-μL pipette tip. The scratch wounds were photographed over a 72 h period using an inverted microscope (Olympus), and the widths of the wounds were quantified using imaging software. Each assay was performed in triplicate.

### Transwell assay

SW480 and HCT-116 cells were respectively transfected with miR-1307 mimics, miR-1307 inhibitors, PRRX1-expression plasmid, or PRRX1 siRNA for 48 h. Then about 1 × 10^4^ SW480-T, SW480-C, SW480, and HCT-116 cells were respectively cultured in a chamber (Corning) with 100 μL serum-free medium, which was put in a 24-well plate containing 600 μL culture medium with 20% FBS. After incubating for 48 h, the cells attached to the lower surface were fixed by 0.4% paraformaldehyde and were stained by 0.1% crystal violet, and then were photographed and counted using the microscope. Each assay was performed in triplicate.

### Xenografts

Animal protocols were approved by the Institutional Animal Care and Use Committee at Soochow University. All animal experiments were complied with the ARRIVE guidelines and were carried out in accordance with the National Institutes of Health guide for the care and use of Laboratory animals (NIH Publications No. 8023, revised 1978). Athymic male SCID mice at about 4-to-6 weeks old were purchased from SLAC int. (Shanghai, China). The mice were randomly divided into groups according to their body weights. SW480-T, SW480-C, PRRX1^oe^ SW480 and PRRX1^oe^ HCT-116 cells were collected and mixed with Matrigel (Corning) at 1:1 ratio by volume. To explore the effect of rs7911488T>C on the growth of CRC, approximately 5 × 10^6^ SW480-T or SW480-C cells were subcutaneously injected in the lower back region of mice (*n* = 8). Tumor volumes were non-blindly measured every 4 days. To explore the effect of PPRX1 on the growth of CRC, approximately 3 × 10^6^ PRRX1^oe^ SW480 or PRRX1^oe^ HCT-116 cells were subcutaneously injected in the lower back region of mice (*n* = 6). Tumor volumes were non-blindly measured every 2 days.

## Results

### rs7911488-T allele contributed to the growth and metastasis of CRC

**Fig. 1:**
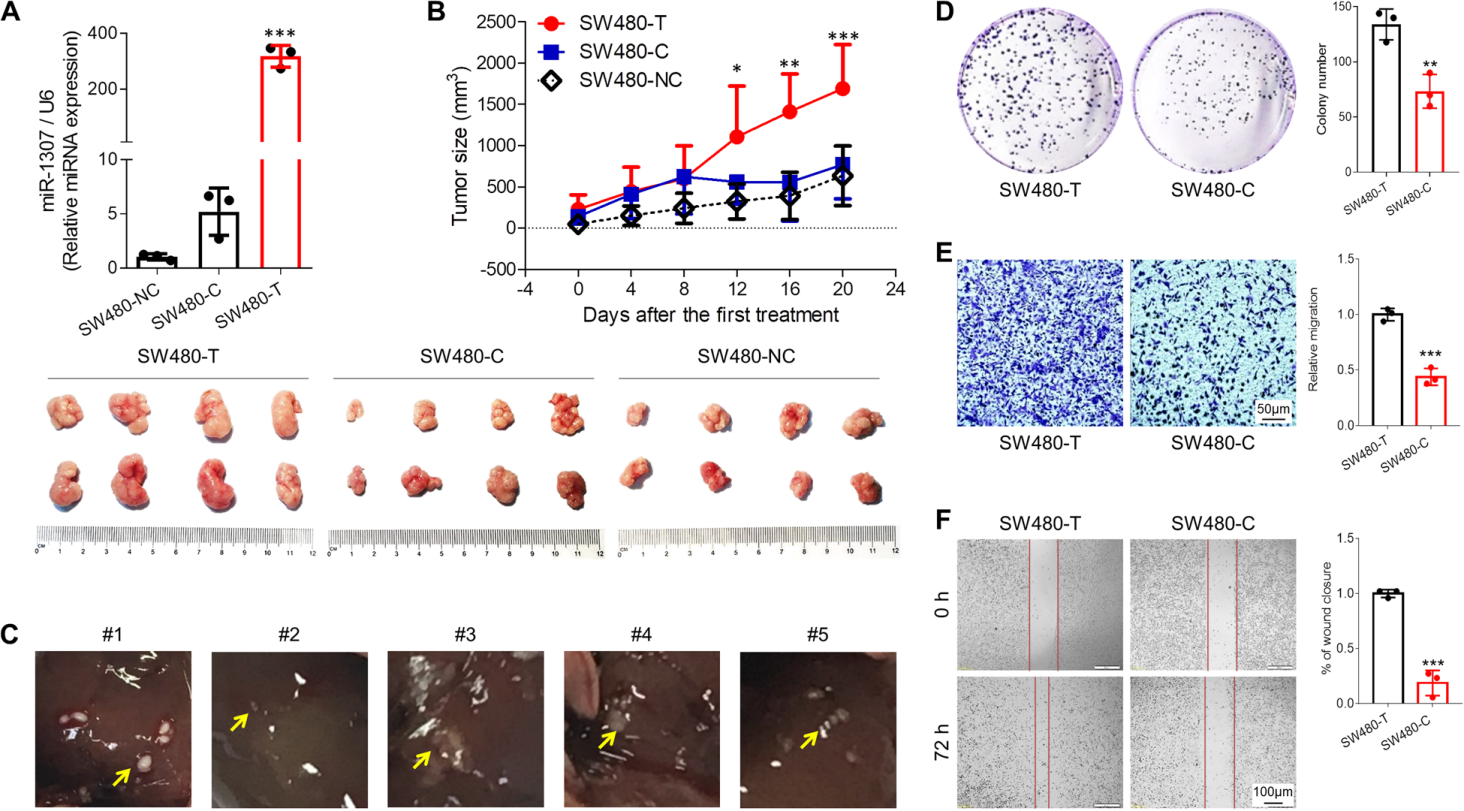
The effects of rs7911488T>C on the growth and metastasis of CRC. **a** miR-1307 expression in SW480-T, SW480-C, and SW480-NC cells. **b** The growth curves and pictures of tumors grafted from SW480-T, SW480-C, and SW480-NC cells in nude mice (*n* = 8). **c** Liver metastases of SW480-T cell xenografts in five nude mice. **d** Colony formation assay of SW480-T and SW480-C cells. **e** Transwell assays of SW480-T and SW480-C cells. **f** Wound healing assays of SW480-T and SW480-C cells. Data represent mean ± SD. Significance was assessed by two-sided *t*-test. ***P < 0.001; **P < 0.01; *P < 0.05.

To explore the roles of rs7911488 T>C in CRC, we generated cell lines for over-expression of rs7911488 T-allelic or C-allelic pre-miR-1307 (SW480-T or SW480-C). The results showed that the expression level of miR-1307 in the SW480-T cells was predominantly higher than the SW480-C cells (Fig. 1a). Then, we subcutaneously grafted SW480-T and SW480-C cells into the lower back of nude mice and found that the growth of SW480-T tumors was obviously faster than SW480-C tumors (Fig. 1b). Furthermore, we observed multiple liver metastases in five of the eight mice inoculated with SW480-T cells (Fig. 1c), but not in the mice inoculated with SW480-C cells. To further confirm the involvement of rs7911488 T>C in the growth and metastasis of CRC, colony formation, transwell assays, and wound healing assays were performed on SW480-T and SW480-C cells. The results showed that the colony counts of SW480-T cells were significantly more than the SW480-C cells (Fig. 1d). Moreover, the numbers of SW480-T cells penetrated the transwell membrane were evidently larger than the SW480-C cells (Fig. 1e). In addition, the scratch widths of SW480-T cells were obviously narrower than the SW480-C cells (Fig. 1f). These findings suggest that rs7911488-T allele contributes to the growth and metastasis of CRC.

### miR-1307 promoted the proliferation and migration of CRC cells

**Fig. 2:**
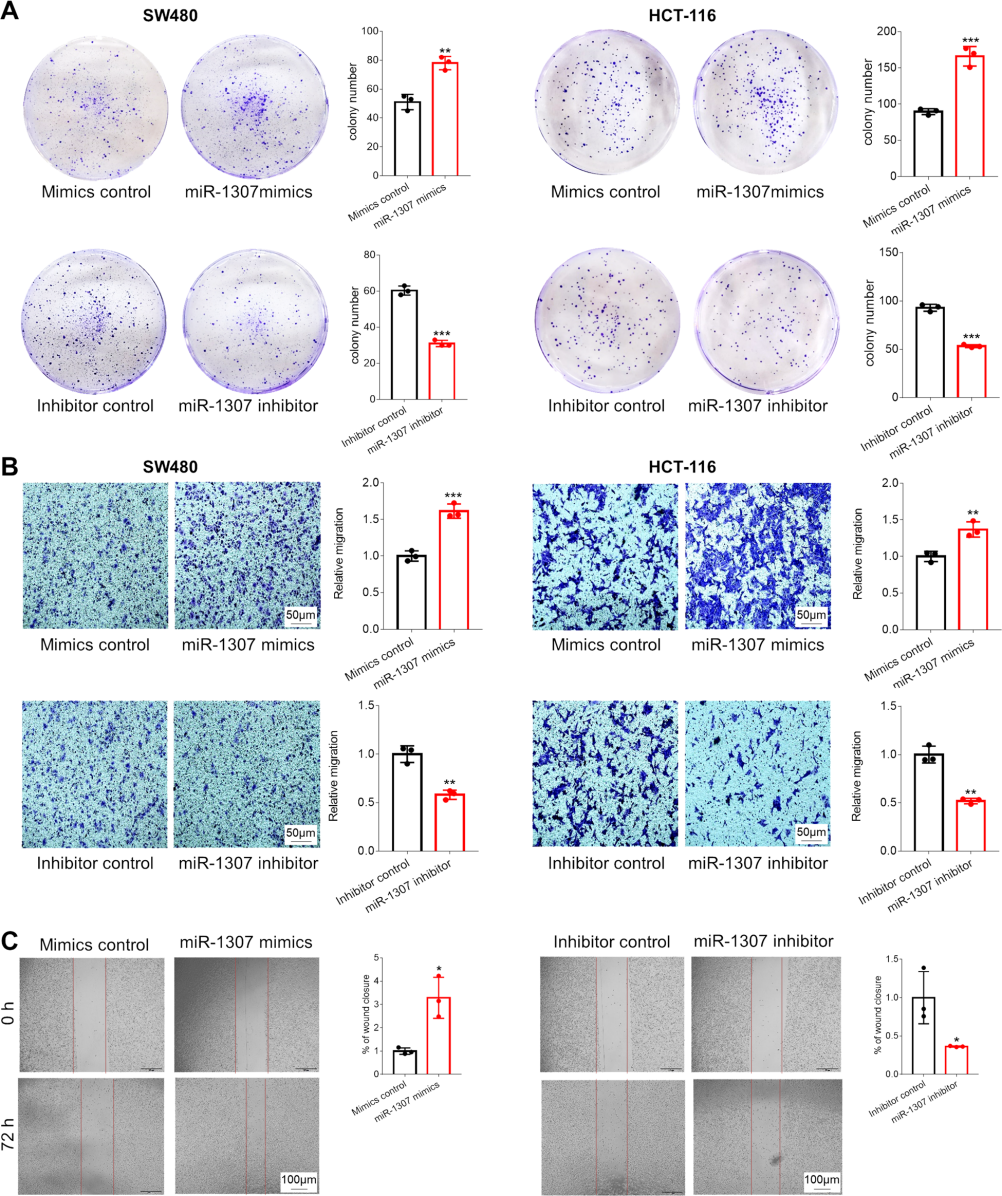
The effects of miR-1307 on the proliferation and migration of SW480 and HCT-116 cells. **a** Colony formation assay to investigate the effects of miR-1307 on the proliferation of SW480 and HCT-116 cells. **b** Transwell assays to investigate the effects of miR-1307 on the migration of SW480 and HCT-116 cells. **c** Wound healing assays to investigate the effects of miR-1307 on the migration of SW480 cells. Data represent mean ± SD. Significance was assessed by two-sided *t*-test. ***P < 0.001; **P < 0.01; *P < 0.05.

Next, we utilized colony formation, transwell assays, and wound healing assays to test the roles of miR-1307 in the proliferation and migration of CRC cells. We transfected miR-1307 mimics or inhibitor into SW480 and HCT-116 cells, respectively, and then detected the numbers of cells formed colony or penetrated the transwell membrane, as well as the widths of scratch wounds. The expression of miR-1307 was evidently enhanced by miR-1307 mimics, but was suppressed by miR-1307 inhibitor in either HCT-116 cells or SW480 cells (Fig. S1). The results demonstrated that the colony numbers of SW480 and HCT-116 cells were significantly increased by miR-1307 mimics, but were reduced by miR-1307 inhibitors (Fig. 2a). The numbers of SW480 and HCT-116 cells penetrated the transwell membrane were also evidently elevated by miR-1307 mimics, but were attenuated by miR-1307 inhibitors (Fig. 2b). Furthermore, the scratch widths of SW480 cells were apparently narrowed by miR-1307 mimics, but were slightly broadened by miR-1307 inhibitors (Fig. 2c). These findings demonstrate that miR-1307 promotes the proliferation and migration of CRC cells.

### miR-1307 inhibited PRRX1 expression in CRC cells

**Fig. 3:**
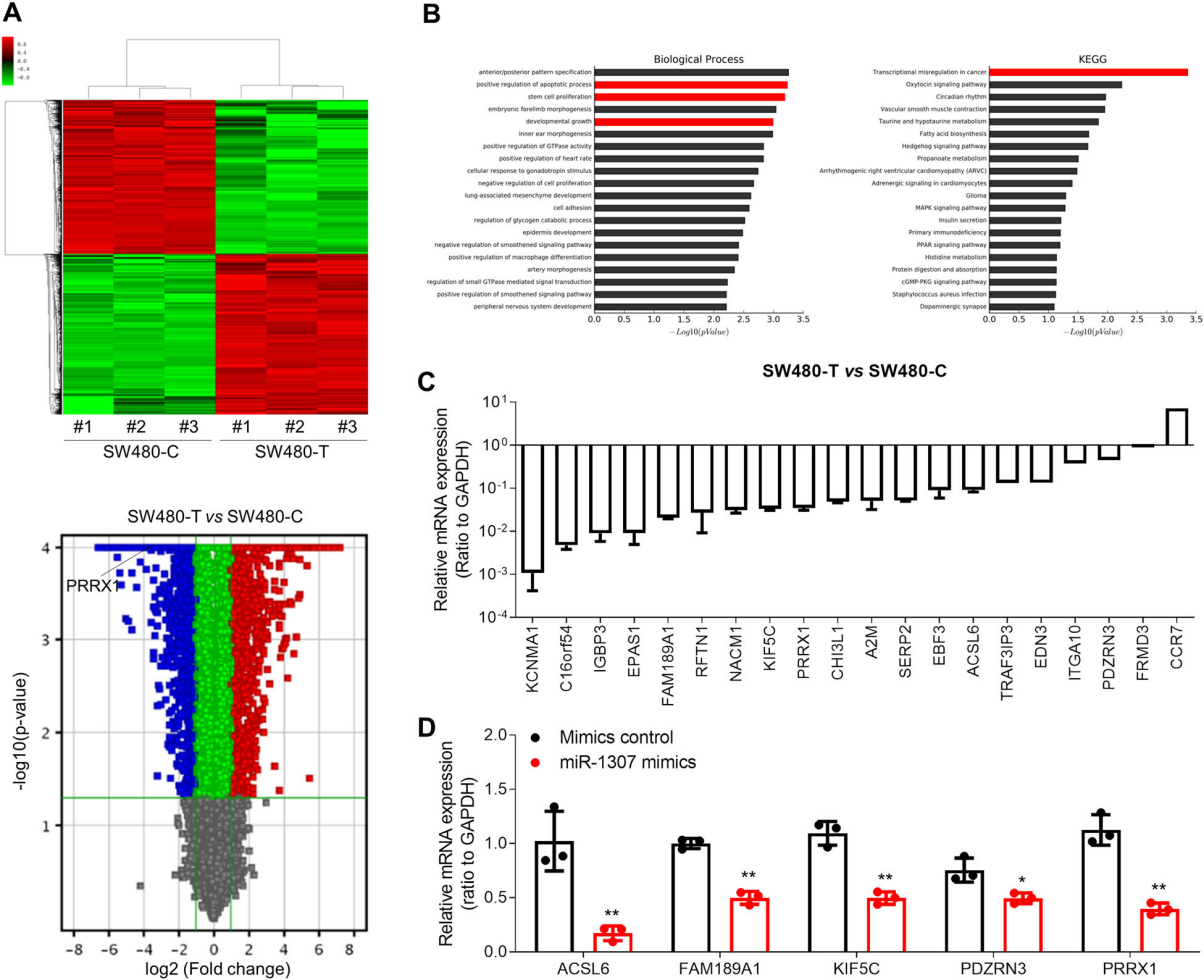
Identification of the target genes for miR-1307. **a** The heatmap and volcano plot of differentially expressed genes in SW480-T and SW480-C cells measured by gene array. **b** GO enrichment analysis and KEGG analysis of the downregulated genes in SW480-T cells. **c** The expression of the downregulated genes (fold change>10 and *P* < 10^−4^) in SW480-T cells relative to SW480-C cells. **d** The inhibitory roles of miR-1307 mimics in the expression of genes in SW480 cells, which were transfected with 50 nM miR-1307 mimics for 48 h. Data represent mean ± SD. Significance was assessed by two-sided *t*-test. **P < 0.01; *P < 0.05.

To explore the mechanism underlying miR-1307-mediated promotion of the proliferation and migration of CRC cells, we firstly detected gene expression in SW480-T and SW480-C cells by gene array. Compared with SW480-C cells, 1387 genes were downregulated and 1449 genes were upregulated in SW480-T cells (Fig. 3a). Then we employed GO enrichment analysis to figure out the biological functions of the downregulated genes. We found that they were mainly related to cell proliferation, development, and apoptosis (Fig. 3b). In addition, KEGG analysis suggested that these downregulated genes were mainly involved in transcriptional regulation in tumors (Fig. 3b). We further verified the expression of tumor-related downregulated genes (*F*c > 10; *P* < 10^−4^) in the SW480-T and SW480-C cells using qPCR analysis. The results showed that the expression levels of these genes in the SW480-T cells were remarkably lower than the SW480-C cells (Fig. 3c), only excepting for CCR7. To further investigate the regulatory roles of miR-1307 in these genes, we transfected miR-1307 mimics into SW480 cells, and then detected the expression of the candidate genes. We found that the expression of five genes including ACSL6, FAM189A1, KIF5C, PDZRN3, and PRRX1 was significantly suppressed by miR-1307 mimics (Fig. 3d). Next, we used the TCGA database to analyze the correlation between these genes and the host gene of miR-1307, USMG5. The results showed that PRRX1 expression was negatively correlated to USMG5 in CRC tissues (Fig. S2). Thus, we tested the regulatory role of miR-1307 in PRRX1 expression.

**Fig. 4:**
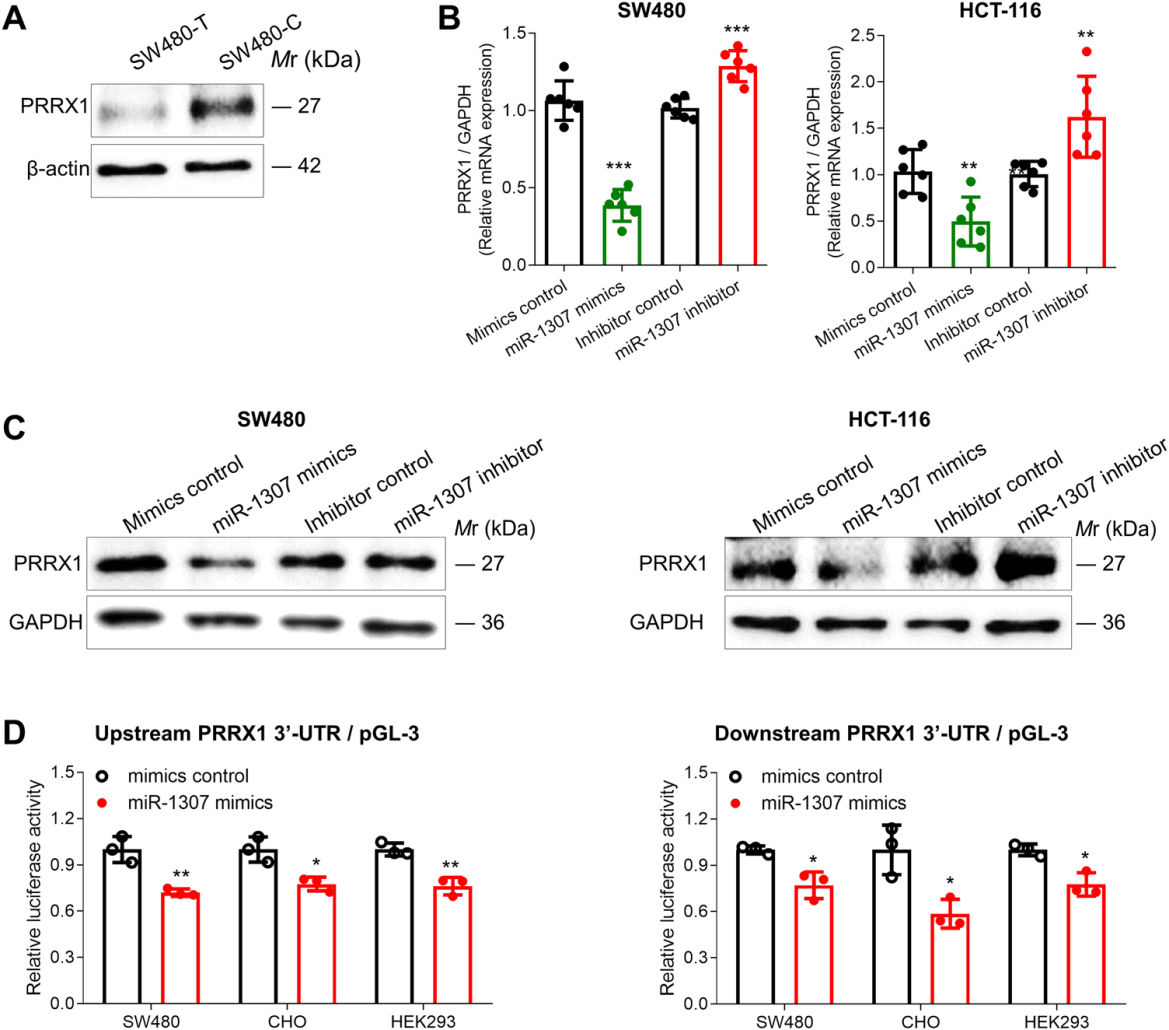
The regulatory mechanism of miR-1307-mediated inhibition of PRRX1. **a** The expression of PRRX1 protein in SW480-T and SW480-C cells. **b** The effect of miR-1307 on the expression of PRRX1 mRNA in SW480 and HCT-116 cells, which were respectively transfected with miR-1307 mimics or inhibitor for 48 h. **c** The effect of miR-1307 on the expression of PRRX1 protein in SW480 and HCT-116 cells, which were respectively transfected with miR-1307 mimics or inhibitor for 72 h. **d** The effect of miR-1307 on the expression activity of PRRX1/3′-UTR/pGL-3 constructs in SW480, CHO, and HEK293 cells. The upstream- and downstream-sequence of PRRX1 3′-UTR were amplified and then cloned into the pGL-3 plasmids. The PRRX1/3′-UTR/pGL-3 constructs were respectively transfected into SW480, CHO, and HEK293 cells with miR-1307 mimics. Data represent mean ± SD. Significance was assessed by two-sided *t*-test. ***P < 0.001; **P < 0.01; *P < 0.05.

We firstly measured the expression level of PRRX1 protein in SW480-T and SW480-C cells. We found that the expression levels of PRRX1 in SW480-T cells were significantly lower than the SW480-C cells (Fig. 4a). Then, we respectively transfected miR-1307 mimics or inhibitor into SW480 and HCT-116 cells, and then measured the expression levels of PRRX1 mRNA and protein. We found that both PRRX1 mRNA and protein were remarkably downregulated by miR-1307 mimics, but were induced by miR-1307 inhibitors in either SW480 or HCT-116 cells (Fig. 4b, c). In addition, luciferase reporter assays were employed to test the binding of miR-1307 to PRRX1 3′-UTR. We generated pGL-3 constructs containing upstream or downstream PRRX1 3′-UTR, and then co-transfected them with miR-1307 mimics into CHO, HEK293, and SW480 cells, respectively. We found that miR-1307 significantly inhibited the expression activity of either upstream or downstream PRRX1/3′-UTR/pGL-3 constructs in CHO, HEK293, and SW480 cells (Fig. 4d). Further investigation showed that miR-1307 inhibited the expression activity of PRRX1/3′-UTR/pGL-3 constructs in a dose-dependent manner (Fig. S3). These findings indicate that miR-1307 suppresses PRRX1 expression by directly binding to PRRX1 3′-UTR with at least two binding-sites.

### PRRX1 inhibited the proliferation and migration of CRC cells

**Fig. 5:**
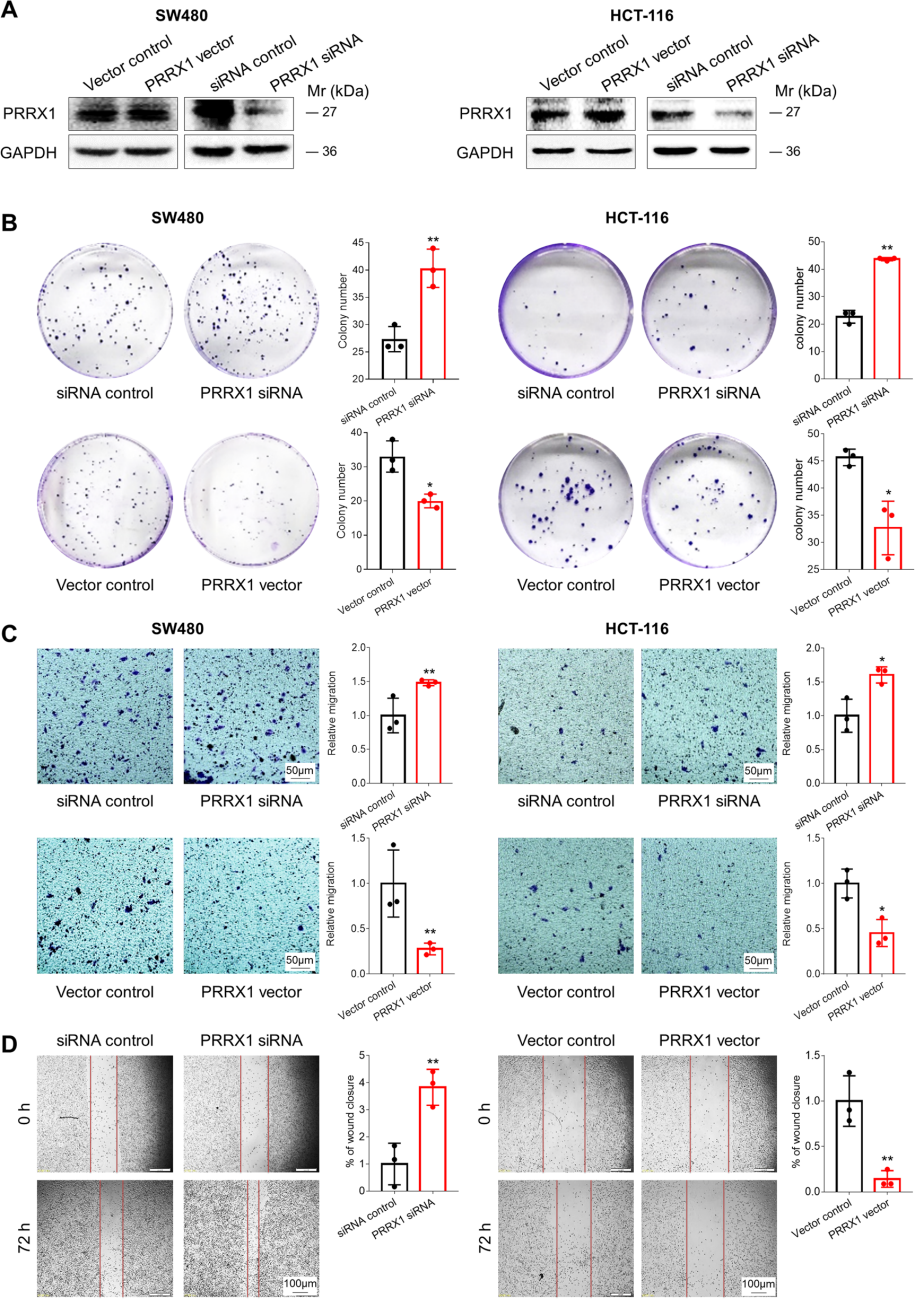
The effect of PRRX1 on the proliferation and migration of SW480 and HCT-116 cells. **a** The expression of PRRX1 protein in SW480 and HCT-116 cells. **b** Colony formation assays for investigating the effects of PRRX1 on the proliferation of SW480 and HCT-116 cells. **c** Transwell assays for investigating the effects of PRRX1 on the migration of SW480 and HCT-116 cells. **d** Wound healing assays for investigating the effects of PRRX1 on the migration of SW480 cells. The SW480 and HCT-116 cells were respectively treated with PRRX1 expression vectors or PRRX1 siRNA for 72 h. Data represent mean ± SD. Significance was assessed by two-sided *t*-test. **P < 0.01; *P < 0.05.

To investigate the influence of PRRX1 on the proliferation and migration of CRC cells, we reinforced and silenced PRRX1 in SW480 and HCT-116 cells by using PRRX1 expression plasmids and siRNA, respectively. WB results demonstrated that PRRX1 expression was enhanced by PRRX1 expression plasmids and was inhibited by PRRX1 siRNA in both SW480 and HCT-116 cells (Fig. 5a). Colony formation assays, transwell assays, and wound healing assays were also performed to test the roles of PRRX1 in the proliferation and migration of CRC cells. We transfected PRRX1 expression plasmids or siRNA into SW480 and HCT-116 cells, respectively, and then detected the numbers of cells formed colony or penetrated the transwell membrane, as well as the widths of scratch wounds. The results showed that the colony numbers of SW480 and HCT-116 cells were significantly elevated by PRRX1 siRNA, but were attenuated by PRRX1 expression plasmids (Fig. 5b). The numbers of SW480 and HCT-116 cells penetrated the transwell membrane were also evidently increased by PRRX1 siRNA, but were decreased by PRRX1 expression plasmids (Fig. 5c). Moreover, the scratch widths of SW480 cells were apparently narrowed by PRRX1 siRNA, and were broadened by PRRX1 expression plasmids (Fig. 5d). These findings demonstrate that PRRX1 inhibits the proliferation and migration of CRC cells.

### PRRX1 involved in miR-1307-promoted proliferation and migration of CRC cells

**Fig. 6:**
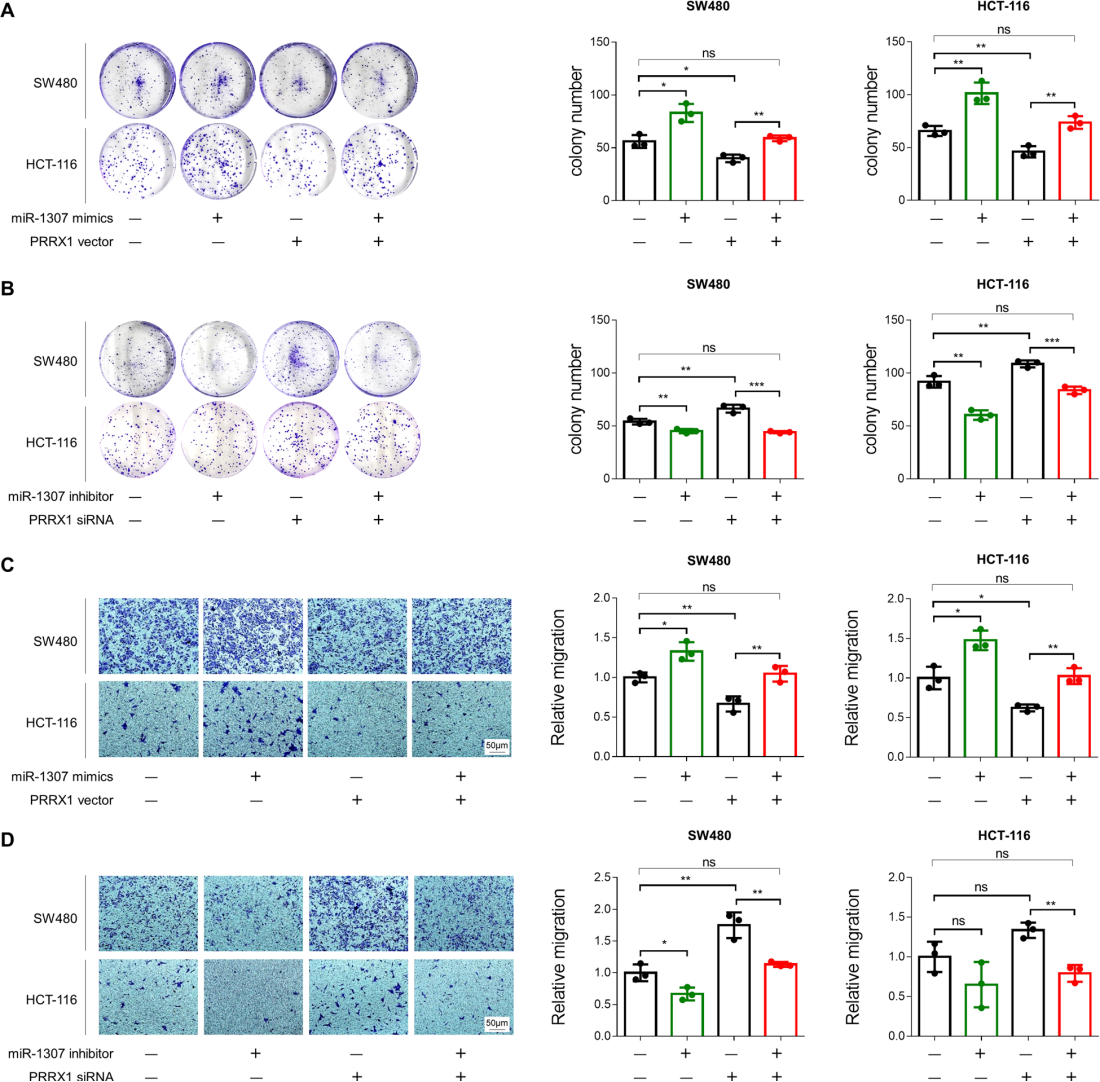
The involvement of PRRX1 in the miR-1307-promoted proliferation and migration of SW480 and HCT-116 cells. **a** Colony formation assays for detecting the proliferative abilities of SW480 and HCT-116 cells, which were treated with miR-1307 mimics and/or PRRX1 expression vectors. **b** Colony formation assays for detecting the proliferative abilities of SW480 and HCT-116 cells, which were treated with miR-1307 inhibitors and/or PRRX1 siRNA. **c** Transwell assays for detecting the migrating abilities of SW480 and HCT-116 cells, which were treated with miR-1307 mimics and/or PRRX1 expression vectors. **d** Transwell assays for detecting the migrating abilities of SW480 and HCT-116 cells, which were treated with miR-1307 inhibitors and/or PRRX1 siRNA. Data represent mean ± SD. Significance was assessed by two-sided *t*-test. ***P < 0.001; **P < 0.01; *P < 0.05; ns, no significance.

Rescue experiments were performed to figure out the involvement of PRRX1 in the miR-1307-promoted proliferation and migration of CRC cells. We transfected miR-1307 mimics with PRRX1 expression plasmids or miR-1307 inhibitors with PRRX1 siRNA into SW480 and HCT-116 cells, respectively, and then detected the numbers of cells formed colony or penetrated the transwell membrane. The results showed that the colony numbers of SW480 and HCT-116 cells were markedly elevated by miR-1307 mimics and were attenuated by PRRX1 expression plasmids, but no significant change was observed for miR-1307 mimics combined with PRRX1 expression plasmids (Fig. 6a). Moreover, the colony numbers of SW480 and HCT-116 cells were markedly reduced by miR-1307 inhibitors and were increased by PRRX1 siRNA, and no significant change was observed for miR-1307 inhibitors combined with PRRX1 siRNA (Fig. 6b). Consistently, the numbers of SW480 and HCT-116 cells penetrated the transwell membrane were evidently elevated by miR-1307 mimics and were attenuated by PRRX1 expression plasmids (Fig. 6c). However, no significant change was observed for miR-1307 mimics combined with PRRX1 expression plasmids (Fig. 6c). The numbers of SW480 and HCT-116 cells penetrated the transwell membrane were also attenuated by miR-1307 inhibitors and were elevated by PRRX1 siRNA, but were not affected by miR-1307 inhibitors combined with PRRX1 siRNA (Fig. 6d). These findings demonstrate that the stimulatory role of miR-1307 mimics and suppressive role of miR-1307 inhibitors in the proliferation and migration of CRC cells were reversed by PRRX1 expression vector and PRRX siRNA, respectively, suggesting that miR-1307 promotes the proliferation and migration of CRC cells through inhibiting PRRX1.

### PRRX1 suppressed the growth of CRC tumors in mice

**Fig. 7:**
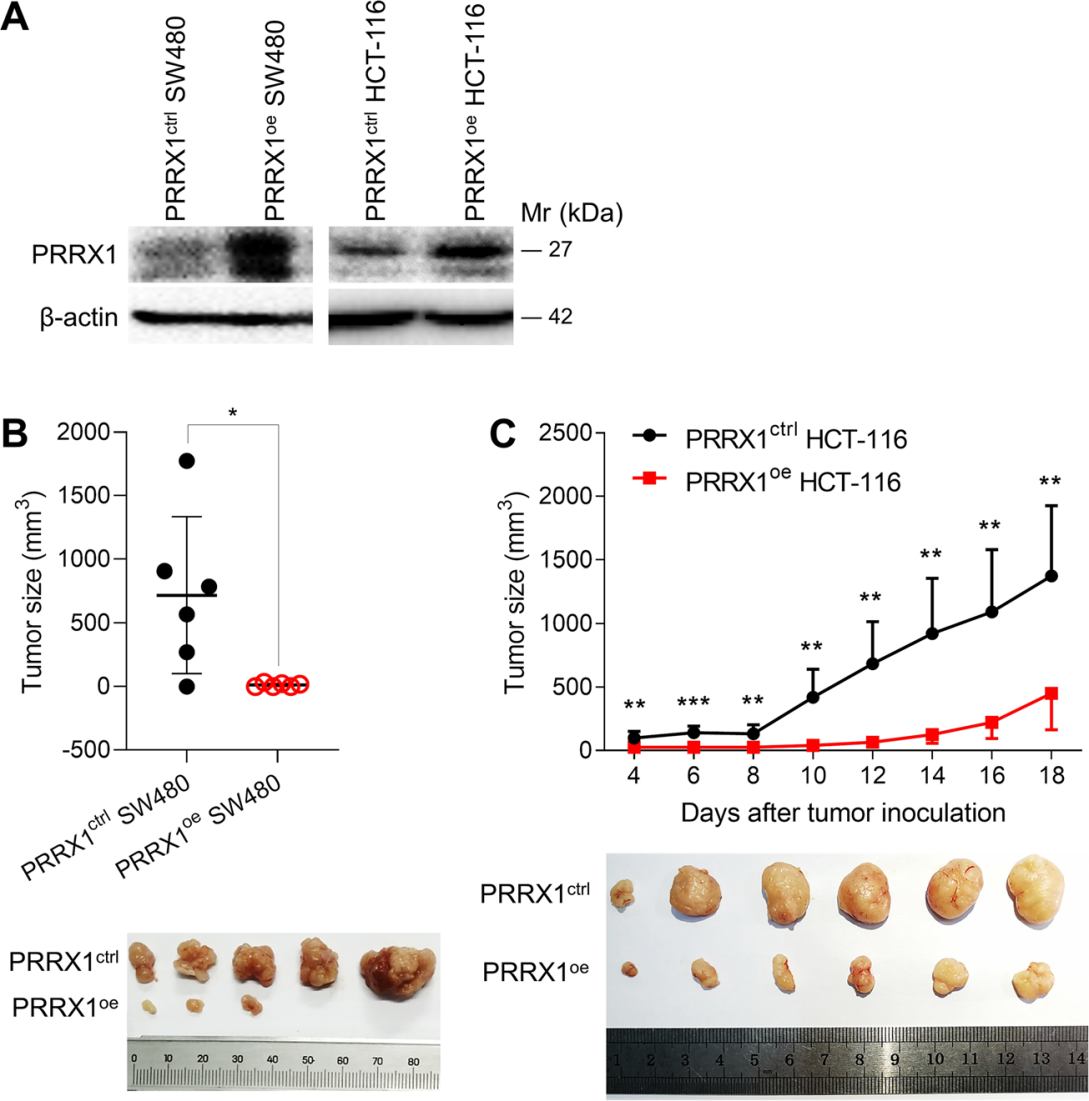
The effect of PRRX1 on the growth of CRC tumors. **a** The expression of PRRX1 protein in PRRX1^oe^ SW480 and PRRX1^oe^ HCT-116 cells. **b** The volumes and photos of tumors grafted with PRRX1^oe^ and PRRX1^ctrl^ SW480 cells in nude mice (*n* = 6). **c** The growth curve and photos of tumors grafted with PRRX1^oe^ and PRRX1^ctrl^ HCT-116 cells in nude mice (*n* = 6). Data represent mean ± SD. Significance was assessed by two-sided *t*-test. ***P < 0.001; **P < 0.01; *P < 0.05.

To further explore the inhibitory role of PRRX1 in tumor growth, we generated cell lines to stably overexpress PRRX1 in SW480 (PRRX1^oe^ SW480) and HCT-116 (PRRX1^oe^ HCT-116) cells. WB results demonstrated that PRRX1 was upregulated in both PRRX1^oe^ SW480, and PRRX1^oe^ HCT-116 cells (Fig. 7a). Then we subcutaneously grafted PRRX1^oe^ SW480 or PRRX1^oe^ HCT-116 cells into the lower back of nude mice. We found that the growth of either PRRX1^oe^ SW480 or PRRX1^oe^ HCT-116 tumors was markedly slower than the controls (Fig. 7b, c). These findings further prove that PRRX1 inhibits the growth of CRC tumors in vivo.

## Discussion

In this study, we discovered that the T allele of the polymorphism rs7911488 in the stem-loop of pre-miR-1307 led to high expression of miR-1307, which inhibited the expression of its target gene PRRX1 and consequently promoted the growth and metastasis of CRC.

Numerous studies have shown that miRNA plays an extremely important role in the occurrence and development of tumors. Moreover, studies have provided evidence to prove that polymorphisms in the miRNA genes (miR-SNPs) can impact the expression or regulatory roles of miRNAs, and consequently participate in tumorigenesis. miR-SNP may disrupt the transcription of pri-miRNA, processing of pri-miRNA and/or pre-miRNA, or miRNA–mRNA interaction^16^. Carlo et al. firstly reported that genetic variants were involved in the development of tumors by altering the processing of mature miRNAs. They found that a mutation in pri-miR-16-1 caused low expression level of miR-16-1 in a family of familial chronic lymphocytic leukemia^27^. A G>A variation at 19 bases in downstream of let-7e led to reduced expression of mature let-7e and was associated with susceptibility to cancers^28^. In addition, rs213210 in pri-miR-219 contributed to increased risk of esophageal cancer^29^, and rs11614913 in pre-miR-196a-2 was correlated to the risk of breast cancer^30^ and lung cancer^31^ in a Chinese population. Our previous studies revealed that rs7911488T>C in the stem-loop of pre-miR-1307 was significantly associated with the susceptibility to CRC by disrupting the maturation of miR-1307^20^. To unreal the roles of rs7911488 in the development of CRC, we generated cell lines for over-expression of rs7911488 T-allelic or C-allelic pre-miR-1307 (SW480-T or SW480-C) and subcutaneously grafted them into the lower back of nude mice. The result showed that the growth of SW480-T tumors, with high expression of miR-1307, was obviously faster than SW480-C tumors. Moreover, the results from the colony formation, transwell assays, and wound healing assays further supported that the proliferative and metastatic abilities of SW480-T cells were more potent than SW480-C cells. These findings demonstrated that rs7911488-T led to increased expression of miR-1307, which promoted the growth and metastasis of CRC.

Next, we employed in vitro experiments to test the hypothesis that miR-1307 can promote the proliferation and migration of CRC cells. Colony formation assays showed that the proliferative ability of SW480 and HCT-116 cells was significantly enhanced by the over-expression of miR-1307, but was weakened by the knockdown of miR-1307. The results of transwell assays and wound healing assays showed that the migration ability of SW480 and HCT-116 cells was significantly boosted by the overexpressed miR-1307, and was reduced by the inhibition of miR-1307. To further investigate the molecular mechanism underlying the miR-1307-promoted proliferation and migration of CRC cells, we utilized several techniques including gene array, qPCR, dual-luciferase reporter assay, statistical analysis based on public database, and WB to figure out the target genes of miR-1307. We firstly detected gene expression in SW480-C and SW480-T cells by gene array, and found that 1387 genes were downregulated and 1449 genes were upregulated in SW480-T cells compared with SW480-C cells. Then, we transferred miR-1307 mimics into SW480 cells and determined the expression of 20 genes dramatically downregulated in the SW480-T cells (fold change > 10 and *P* < 10^−4^) using qPCR. We found that 5 genes were significantly downregulated by miR-1307. Based on the analysis of TCGA data, we found that the host gene of miR-1307, USMG5, was negatively correlated with PRRX1 expression. Thereafter, we employed loss- and gain-of-function approaches to prove that miR-1307 inhibited PRRX1 mRNA and protein in CRC cells. Additionally, dual-luciferase reporter assays were used to confirm that miR-1307 directly bond to PRRX1 3′-UTR. These findings provide evidence to support the conclusion that PRRX1 is a target gene of miR-1307. Up to now, several important genes involved in tumorigenesis had been identified as the target genes of miR-1307, such as BCL2^20^, TYMS^21^, MDM4^32^, SMYD4^23^, DAB2IP^24^, FOXO3A^25^, ING5^26^, and ISM1^33^, indicating critical roles of miR-1307 in cancers.

From the observations of rescue experiments, we can draw a conclusion that miR-1307 promotes the proliferation and migration of CRC cells through inhibition of PRRX1. The results from the colony formation, transwell assays, and wound healing assays showed that miR-1307-promoted proliferation and migration of CRC cells could be reversed by PRRX1 over-expression, while the inhibitory roles of miR-1307 inhibitor in the proliferation and migration of CRC cells could also be eliminated by PRRX1 knockdown. Emerging evidence suggest that miR-1307 contributes the occurrence and development of tumors. For instance, high expression of miR-1307 was detected in the serum of breast cancer patients, which significantly promoted the proliferation of breast cancer cells^22^. miR-1307 can also accelerate the progression of breast cancer by inhibiting the expression of SMYD4^23^. High expression of miR-1307 was also measured in liver cancer tissues, and was significantly associated with poor prognosis of patients. Moreover, miR-1307 contributed to the proliferation and metastasis of liver cancer cells by suppressing DAB2IP^24^. Additionally, studies have shown that miR-1307 is overexpressed in prostate cancer cells and ovarian cancer cells, and promotes cancer cell proliferation by targeting FOXO3A^25^. These findings demonstrate that miR-1307 exerts its roles in various cancers by regulating multiple target genes.

PRRX1, an important member of the paired family of homeobox proteins, locates in chromosome 1q24. PRRX1 is a transcription activator and contains an OAR domain and a homeobox DNA-binding domain. The homeobox DNA-binding domain is a 60-amino-acid motif that binds to DNA via a helix-turn-helix structure, thereby playing a role in transcriptional regulation of gene expression. PRRX1 is abnormally expressed in many diseases and is involved in tumor metastasis, bone maturation, liver fibrosis, cardiovascular diseases, and adipogenesis. Especially, it is closely correlated to the occurrence of epithelial-mesenchymal transition (EMT) and stemness maintenance of tumor stem cells, consequently affecting the proliferation and metastasis of cancer cells^34,35^. In this study, we investigated the effects of PRRX1 in the proliferation and migration of CRC cells in vivo and in vitro. Contrary to miR-1307, PRRX1 over-expression in SW480 and HCT-116 cells markedly reduced the proliferative and migrating abilities of the cells. However, knockdown of PRRX1 in SW480 and HCT-116 cells apparently stimulated the proliferation and migration of the cells. Furthermore, in vivo experiments demonstrated that the growth of either SW480 or HCT-116 cells in nude mice was obviously suppressed by PRRX1 over-expression, indicating that PRRX1 can inhibit the growth of CRC cells. Interestedly, the effect of PRRX1 over-expression on the growth of either SW480 or HCT-116 cells in vivo is obviously more potent than that in vitro. This might because that the change fold of the expression level of PRRX1 in either PRRX1^oe^ SW480 or PRRX1^oe^ HCT-116 cells (Fig. 7a) is apparently larger than that in the cells transfected with PRRX1 vector (Fig. 5a). Also, since PRRX1 is a transcription activator, the regulation of PRRX1 on its target genes is sustained and long-lasting in the stable cell lines, but not in the transiently transfected cells. A previous study showed that PRRX1 was downregulated in liver cancer cells and was positively correlated with the expression of the tumor suppressor gene p53, which inhibited the apoptosis of liver cancer cells. Meanwhile, the overall survival of liver cancer patients with low expression of PRRX1 and p53 was significantly shortened^36^. However, contradictory roles of PRRX1 have also been reported in the other types of tumors. In 2012, Ocana firstly discovered that PRRX1 promoted the EMT of tumor cells and was associated with tumor metastasis^37^. In breast cancer, PRRX1b regulates EMT through the Wnt/β-catenin signaling pathway, consequently leading to invasion and metastasis of cancer cells^38^. In gastric cancer, PRRX1 stimulates translocation of β-catenin into the nucleus upon Wnt pathway activation, thereby inducing c-Myc and consequent EMT of tumor cells^39^. In head and neck squamous cell carcinoma cells, PRRX1 has a synergistic effect with the activated TGF-β1 on the promotion of the migration and invasion of cancer cells, and regulates the phenotypic plasticity and dormancy of tumor cells^40^. These findings suggest that PRRX1 plays divergent roles in different types of tumors.

The 3′-UTR of PRRX1 gene contains 3391 nucleotides, suggesting that its expression may be likely regulated by miRNAs. Indeed, we found that PRRX1 expression was regulated by miR-1307. Furthermore, several miRNAs have been identified to regulate the expression of PRRX1. In squamous cell carcinoma, PRRX1 expression is evidently negatively correlated with miR-642b-3p, which is downregulated in PRRX1-overexpressed cells. Reinforced expression of miR-642b-3p can reverse the PRRX1-promoted EMT and cell proliferation^40^. In lung cancer, PRRX1 knockdown inhibits the expression of Caspase 3, caspase 9, Apaf-1, and cytochrome C, leading to enhanced anti-apoptotic capacity and resistance to cisplatin in lung cancer cells^41^. In CRC, miR-106b suppresses the proliferation and metastasis of tumor cells by inhibiting PRRX1^42^. Meanwhile, TGF-β1 reduces the expression of miR-106b, and consequently elevates the expression of PRRX1, thereby promoting the occurrence of EMT^42^. In triple-negative breast cancer, the expression of PRRX1 is downregulated by miR-655, which consequently suppresses the occurrence of EMT^43^. These findings suggest that the expression of PRRX1 is widely regulated by multiple miRNAs in cancers.

Each miRNA can regulate multiple target genes. Our results from the gene array analysis demonstrated that thousands of genes were regulated by miR-1307. In the current study, we provided the evidence to prove that PRRX1 is a direct target gene of miR-1307. The other target genes of miR-1307 involved in the promotion of the proliferation and metastasis of CRC cells remain to be further studied. Although we have elucidated the molecular mechanism underlying miR-1307-promoted the proliferation and metastasis of CRC cells by inhibiting PRRX1, the regulatory mechanisms of PRRX1 in the inhibition of the proliferation and metastasis of CRC cells are still unclear. In addition, whether miR-1307/PRRX1 axis is a therapeutic target needs further investigation.

In summary, our study uncovered a novel etiological mechanism entailing rs7911488 T allele-promoted the proliferation and metastasis of CRC cells through miR-1307/PRRX1 axis. The T allele of rs7911488 leads to high expression of miR-1307 and consequent low-expression of PRRX1, which promotes the proliferation and metastasis of CRC cells. Moreover, PRRX1 is a vital gene in EMT process and plays an extremely important role in tumor proliferation and metastasis. Thus, our findings provide new insights into the involvement of genetic variants in the development of CRC and may offer potential targets for cancer therapy.

## Supplementary information

Supplementary Tables

Supplementary Figure Legends

Figure S1

Figure S2

Figure S3

## Data Availability

The data that support the findings of this work are obtainable from the corresponding author based on reasonable request.
