# Supplementary material for: The rs7911488-T allele promotes the growth and metastasis of colorectal cancer through modulating miR-1307/PRRX1
Source: Cell Death Dis. 2020 Aug 7;11(8):651. doi: 10.1038/s41419-020-02834-x (PMC7434880; doi:10.1038/s41419-020-02834-x)
Supplement: Supplementary file 1 — Supplementary Tables [file 41419_2020_2834_MOESM1_ESM.docx]

**Supplementary Tables**

**Table S1.** Primers used for qPCR analysis.

| Gene | Forward primer (5’→3’) | Reverse primer (5’→3’) |
| --- | --- | --- |
| A2M | ACCAGGGCAGACAGTGAAAT | GTGCTCTGTCCTTCCACCTG |
| ACSL6 | TGTCGTCTATTGCCACGGAG | CCACCAAGACTGGCCTGAAT |
| C16orf54 | CCAGTTTCCCAGAGCCACAA | TGAGACGTGTGCAGATGGAG |
| CCR7 | CCCTTGGGTGTCAAAGGTAAA | AAACTGATGCGTGAAGTGCTG |
| CHI3L1 | GCAACACTGACTATGCTGTGG | GAGTGAAGCTCCTCCCGAAG |
| EBF3 | GTGTGGAGCGAGCTGATAACT | GGATCACCCGGATGTCTTGG |
| EDN3 | ATATGACAAGGCCTGCCTGC | GGTCCTTGACTTCAACCTCCTTT |
| EPAS1 | GGGACTTACACAGGTGGAGC | GACCGTGCACTTCATCCTCA |
| FAM189A1 | CTGCCAGCCAGGTTACAAGT | GCCAGGATCCTCTGGAGACA |
| FRMD3 | CCTCACCCATGCAAGGATTC | AGGCAGCTGGTGTTGAAGTA |
| IGFBP3 | CAGAATATGGTCCCTGCCGT | GGCGTCTACTTGCTCTGCAT |
| ITGA10 | TAATGGTGGCCTGCACAGAA | CACTCTTCCAGCCTCACAGG |
| KCNMA1 | TGGCCTCCTCCATGGTGA | TTCTGGGCCTCCTTCGTCT |
| KIF5C | ATGGCTGGATAACTGCCGAAG | CTGCAGTACACAGGACACACT |
| NCAM1 | GGGAGATCAGCGTTGGAGAG | AGCTTCTGAAAGATCTTCACGTT |
| PDZRN3 | AGTCTGACTCTTGTCCTGCATC | CTGCCGTTGACCTCAATAATCC |
| PRRX1 | TGATGCTTTTGTGCGAGAAGA | AGGGAAGCGTTTTTATTGGCT |
| RFTN1 | CCTGGAAGGTGTCGAAGTG | TCTGCCTGTTGTGCATTTCTTC |
| SERP2 | TGTTGTCTGTGGCTCAGCTAT | CGGATGCACCAAGATGTCCT |
| TRAF3IP3 | TCCACAGGACACTCCCATCA | TAAGCTGTCCCTTCCAAGAGC |
