# Supplementary material for: The rs7911488-T allele promotes the growth and metastasis of colorectal cancer through modulating miR-1307/PRRX1
Source: Cell Death Dis. 2020 Aug 7;11(8):651. doi: 10.1038/s41419-020-02834-x (PMC7434880; doi:10.1038/s41419-020-02834-x)
Supplement: Supplementary file 2 — Supplementary Figure Legends [file 41419_2020_2834_MOESM2_ESM.docx]

**Supplementary Figure Legends**

**Figure S1.** The effect of miR-1307 mimics and inhibitor on the expression of miR-1307 in SW480 and HCT-116 cells.

**Figure S2.** The correlation of the expression of miR-1307 host gene (USMG5) with ACSL6, FAM189A1, KIF5C, PDZRN3, and PRRX1 in the TCGA samples.

**Figure S3.** The effect of miR-1307 mimics on the expression activity of PRRX1/3'-UTR/pGL-3 constructs in SW480 cells.
