# Supplementary figures and images for: The rs7911488-T allele promotes the growth and metastasis of colorectal cancer through modulating miR-1307/PRRX1
Source: Cell Death Dis. 2020 Aug 7;11(8):651. doi: 10.1038/s41419-020-02834-x (PMC7434880; doi:10.1038/s41419-020-02834-x)

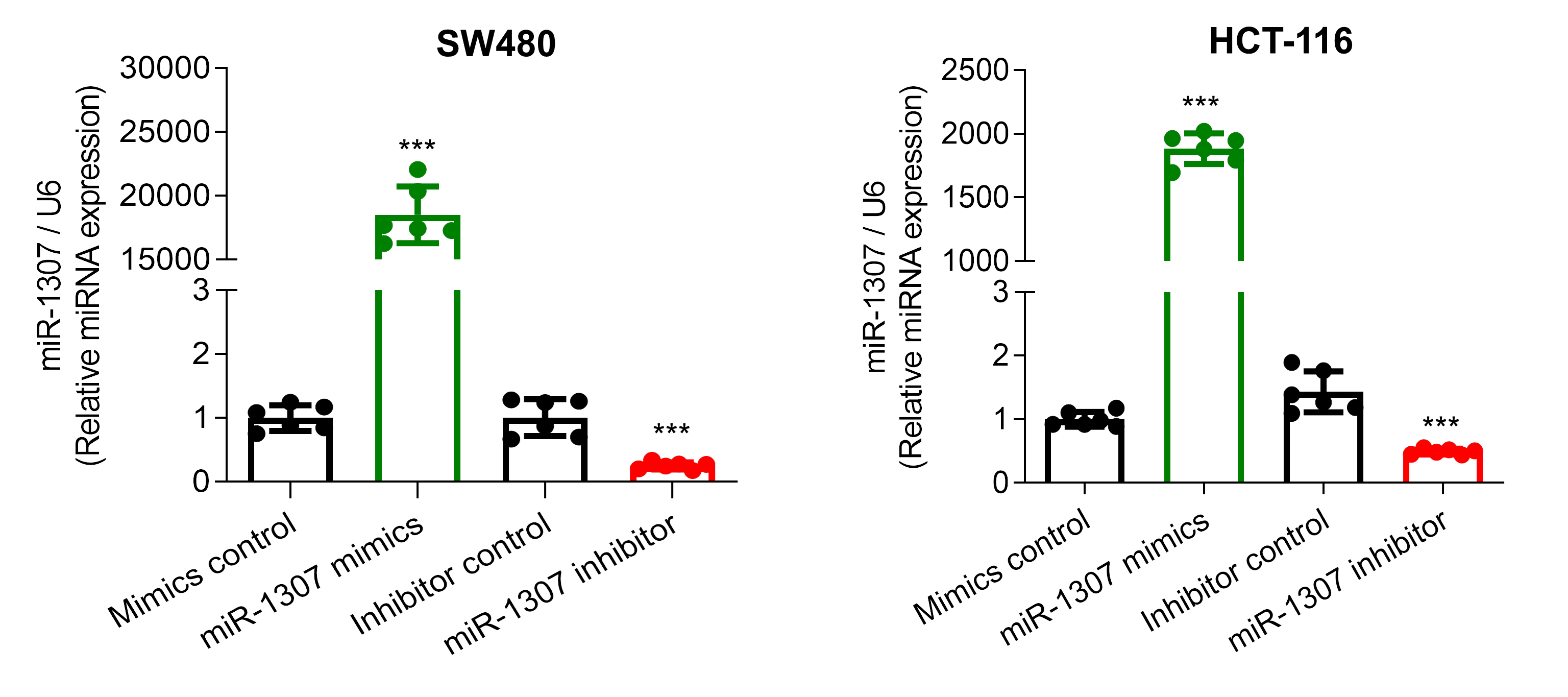

Supplement: Supplementary file 3 — Figure S1 [file 41419_2020_2834_MOESM3_ESM.tif]

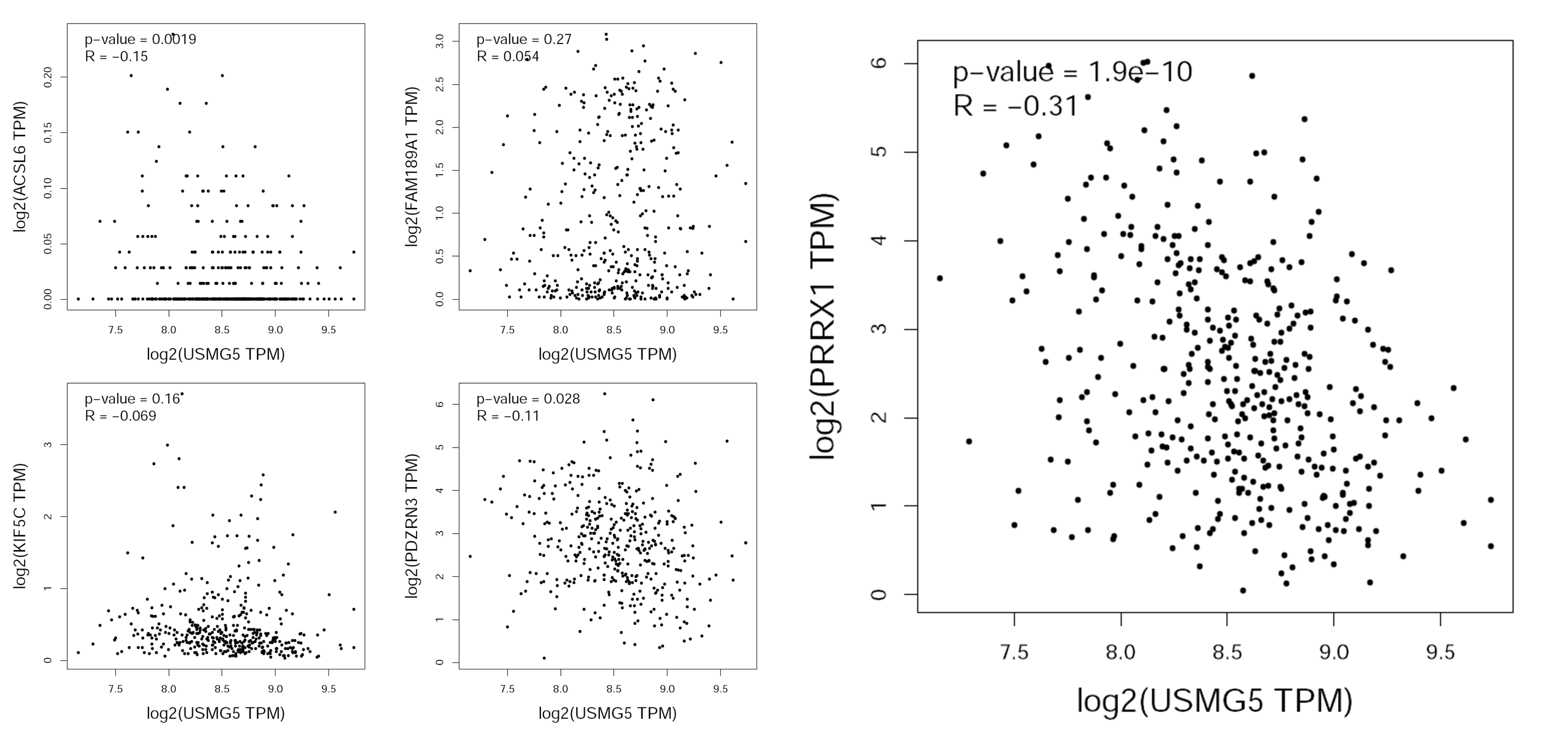

Supplement: Supplementary file 4 — Figure S2 [file 41419_2020_2834_MOESM4_ESM.tif]

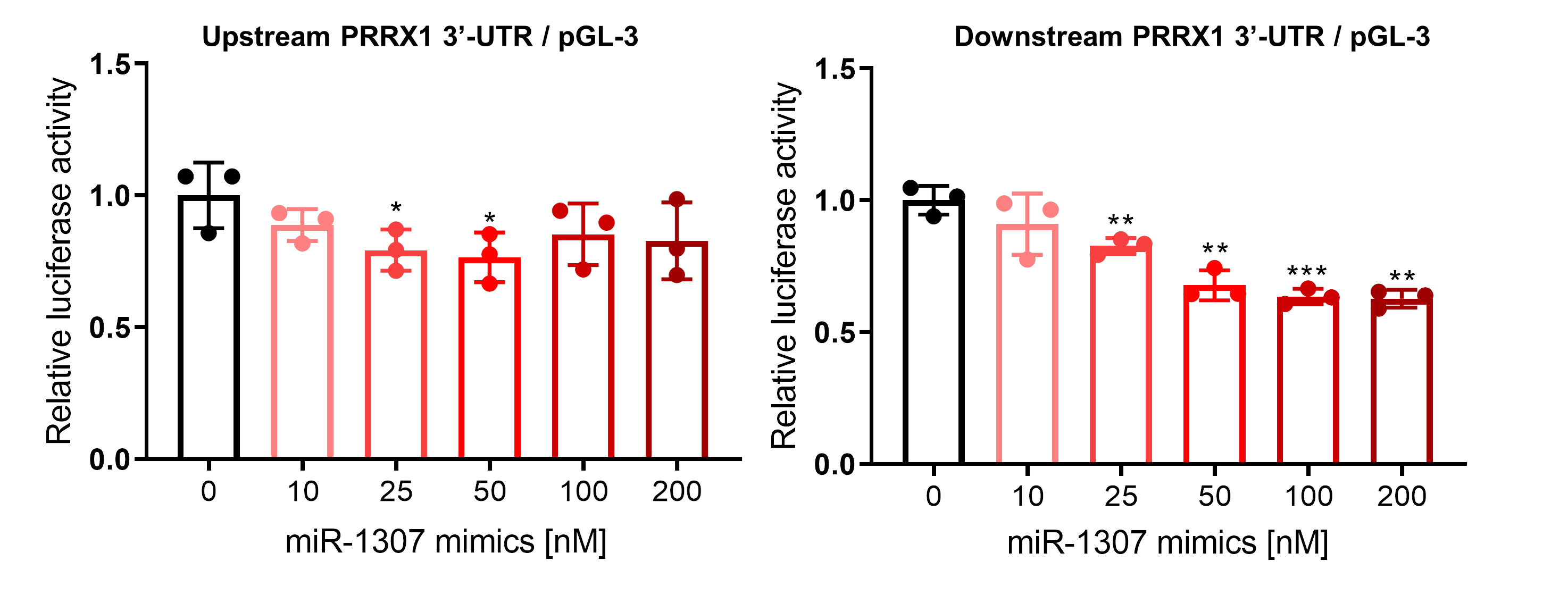

Supplement: Supplementary file 5 — Figure S3 [file 41419_2020_2834_MOESM5_ESM.tif]
